# Advances in Laryngoscopy

**DOI:** 10.12688/f1000research.7045.1

**Published:** 2015-12-08

**Authors:** Michael Aziz

**Affiliations:** 1 Department of Anesthesiology & Perioperative Medicine, Oregon Health & Science University, Porltand, Oregon, 97239, USA

**Keywords:** Laryngoscopy, Optical Stylets, Flexible bronchoscopy, Video laryngoscopy, Airway management

## Abstract

Recent technological advances have made airway management safer. Because difficult intubation remains challenging to predict, having tools readily available that can be used to manage a difficult airway in any setting is critical. Fortunately, video technology has resulted in improvements for intubation performance while using laryngoscopy by various means. These technologies have been applied to rigid optical stylets, flexible intubation scopes, and, most notably, rigid laryngoscopes. These tools have proven effective for the anticipated difficult airway as well as the unanticipated difficult airway.

## Introduction

Airway management remains a difficult skill to master. Competency requires didactic instruction as well as hands-on training with simulators and human patients. To augment challenges with training, airway management is burdened with poor prediction models for difficulty or failure. Indeed, most studies of bedside tests may only marginally predict that direct laryngoscopy fails to achieve an adequate laryngeal view. These tests have very limited application to actual intubation success rates and tools other than direct laryngoscopy with a Macintosh blade. Fortunately, recent advances in airway management have facilitated easier intubation by augmenting the laryngeal view with video technologies. Application of light-emitting diode (LED) light, liquid crystal display (LCD) screens, and complementary metal–oxide–semiconductor (CMOS) video chip technology has made video augmentation more portable, easier to use, and feasible in today’s economic climate. This review will briefly discuss how video technology has changed optical stylets and flexible intubation scopes. Further emphasis will be placed on newer-generation rigid video laryngoscopes.

## Optical stylets

Older optical stylets used fiberoptic bundles and thus required an eyepiece that the provider would have to place their eye on. Today’s optical stylets use video chips and thus can incorporate an easy-to-view video screen on the handle itself. This simplicity and ease of use result in improvements in intubation performance, especially when the neck is mobilized ^1^. Other newer optical stylets have also incorporated technologies with flexible tips ^2^. These attached video screens have overcome a classic barrier to optical stylets—that the head and eye of the provider need to follow a moving target as the scope is advanced through the pharynx and redirected toward the trachea. Instead, a screen that swivels allows the provider to remain in a neutral position to facilitate laryngoscopy. Furthermore, the use of a video camera provides a much wider field of view than the narrow field of view provided by fiberoptic devices.

## Flexible intubation scopes

Flexible intubation scopes have done away with an old name of “flexible fiberoptic” as the optical component no longer utilizes fiberoptic bundles. Today’s technologies also use a video chip carried on the distal end of the bronchoscope. As flexible intubation remains the standard tool for the anticipated difficult airway, better optics and fewer degradation problems related to fiberoptic bundle fracture stand to improve the reliability of awake flexible bronchoscopic airway management. The literature has yet to offer comparisons of video bronchoscopic techniques with older fiberoptic techniques. However, these technologies have allowed them to become more portable. They do not require a large cart with a light source and video processor.

In recent years, bronchoscopes have become more portable and have even come in disposable forms. A disposable approach permits availability of this gold-standard airway management tool in areas where difficult airways are managed less frequently, such as an ambulatory surgery center. Although the technology is not quite as robust as a traditional platform bronchoscopic system, it can be effectively used for difficult airway management ^3, 4^.

## Video laryngoscopy

Video laryngoscopy made its introduction to airway management many years ago with the introduction of the GlideScope video laryngoscope (Verathon, Seattle, WA, USA). Since that time, we have learned much about the utility of the devices and have generated questions to guide future investigation. These tools have been shown to consistently provide an improved view of the larynx compared with direct laryngoscopy. Research has attempted to identify whether these benefits translate to an improvement in actual intubation difficulty or success rate, or both. As success rates for tracheal intubation using direct laryngoscopy in experienced hands are very high, there does not seem to be added benefit beyond improvement of laryngeal view for the undifferentiated airway across age spectrums ^5, 6^. However, evidence has made it clear that video laryngoscopy eases intubation difficulty and increases first-attempt success rates in the airway predicted to be difficult to intubate by direct laryngoscopy ^7– 10^. These benefits are seen for patients who are obese, have a raised Mallampati score, or have reduced cervical motion from pathology or cervical spine precautions.

## Video laryngoscopy: the novice provider

Video laryngoscopy offers significant benefits for the provider who is less experienced with airway management. Compared with direct laryngoscopy, intubation success rates are higher with video laryngoscopy in the hands of novice providers ^11, 12^. These benefits come both with devices that are video-assisted direct laryngoscopes and with those with acutely curved indirect laryngoscopes. An instructor-guided laryngoscopy with a video device appears to accelerate even direct laryngoscopy skills ^13^.

## Video laryngoscopy: awake airway management

Compared with awake fiberoptic intubation, awake video laryngoscopy has been evaluated in the potential difficult airway ^14^. Findings demonstrated similar performance with both techniques. However, validity of the study findings is limited by sedation of study patients, exclusion of those with neck pathologies, and inappropriate post-randomization exclusions ^15^. In another study of awake nasal intubation with flexible bronchoscopy versus video laryngoscopy, techniques performed similarly ^16^. In this study, patients in both groups were sufficiently sedated to avoid recall of the procedure. As such, findings may not apply to the true “awake” intubation. Despite these limitations, awake video laryngoscopy may be a tool that is easier to learn and master than flexible bronchoscopic intubation and likely has a future role in awake airway management.

## Video laryngoscopy: recording and archiving

One of the potential benefits of video technology is the capacity to record and archive still images or video clips from the laryngoscopy. These recordings can be used to confirm tracheal tube placement, document laryngeal view, document absence of trauma, and teach future laryngoscopists. Today’s anesthesia record contains a narrative describing the device, laryngeal view on the Cormack and Lehane scale, absence of trauma, and confirmation of tube placement with various tools. The future electronic record could feasibly store a single picture or video clip that tells the airway management story. To date, anesthesia information management systems have not incorporated archiving of digital airway images as part of the anesthesia record, but this future seems feasible. This advancement is poised to change how we define difficult airway management. Instead of describing a laryngeal view by Cormack-Lehane grading, a video recording may better tell the story and also better describe challenges related to tube passage that may be difficult to describe in a narrative.

## Video laryngoscopy: outside of the operating room

Early investigations in various clinical environments outside of the operating room suggest potential benefit. These environments contain both difficult airway scenarios and providers with less airway management experience than is often found in the operating room. In critical care and emergency medicine environments, video laryngoscopy was associated with a higher intubation success rate in patients with predictors of difficult direct laryngoscopy ^10, 17– 19^. There is one randomized trial to confirm benefit of video laryngoscopy in the critical care environment ^20^. The findings are impressive, but interpretation of the results is limited by a select patient and provider population. In the setting of trauma, the use of video laryngoscopy was associated merely with longer intubation times ^21^. In the obstetric environment, video laryngoscopy has been used for emergency airway management, potential difficult intubations, and rescue of failure of direct laryngoscopy ^22^. Finally, in prehospital emergency medicine, video laryngoscopy is associated with a reduction in the number of intubation attempts and shorter laryngoscopy time than direct laryngoscopy ^23, 24^. However, these studies are observational in nature and interpretation of the results deserves some caution because of the possibility of confounding factors. Prospective randomized studies are much more difficult to perform in these dynamic and emergent environments. Clinical care appears to be transitioning to primary use of video laryngoscopy in these environments, but the literature has not yet confirmed that this practice should be standard in these environments. Prospective randomized controlled trials are still needed to confirm the potential benefit for all airway management within each of these environments.

## Video laryngoscopy: as a rescue device

When direct laryngoscopy fails, it is unclear how to proceed with airway management. The American Society of Anesthesiologists’ difficult airway algorithm calls for transitioning to alternate airway management techniques, but it is not clear which techniques are optimal ^25^. Current intubation rescue techniques include video laryngoscopy, flexible bronchoscopic intubation, use of a lighted stylet, or insertion of a supraglottic airway as a conduit to tracheal intubation. One study across two centers demonstrated that rescue using the GlideScope video laryngoscope was successful in 94% of cases (224/239) after failed direct laryngoscopy ^26^. The Pentax AWS (Ambu, Ballerup, Denmark) was found to achieve successful intubation in 99% of cases (268/270) in which direct laryngoscopy failed to achieve an adequate laryngeal view for intubation ^27^. In a large observational study of a new algorithm involving a small group of providers and select group of patients, failed direct laryngoscopy was rescued by using the Airtraq system (Prodol Meditec SA, Getxo, Spain) in 27 out of 28 cases ^28^. Others reported that when mask ventilation and intubation are both difficult, supraglottic airways restore ventilation in 94% of cases (16/17) ^29^. In summary, video laryngoscopy appears to have a promising role in the management of failed direct laryngoscopy as a rescue technique with a high success rate.

## Video laryngoscopy: difficulty or failure

Despite these many benefits for the difficult airway, video laryngoscopy can fail. One source of failure is an inability to achieve a laryngeal view. However, a frequent and more perplexing scenario is that of an adequate laryngeal view but inability to pass the tube into the trachea. This difficulty may occur with acutely curved indirect laryngoscopes, channeled video laryngoscopes, or even video-assisted direct laryngoscopes. A recent study identified predictors of failure with the GlideScope video laryngoscope ^26^. The strongest predictor for failure is neck pathology from tumor, radiation, or surgical scar. This evidence suggests that flexible techniques remain an important tool to master and maintain competency with. Indeed, awake airway management with flexible techniques remains the gold standard for difficult airway management in the cooperative patient for experienced providers. The key challenge is to maintain this competency.

**Figure 1.**
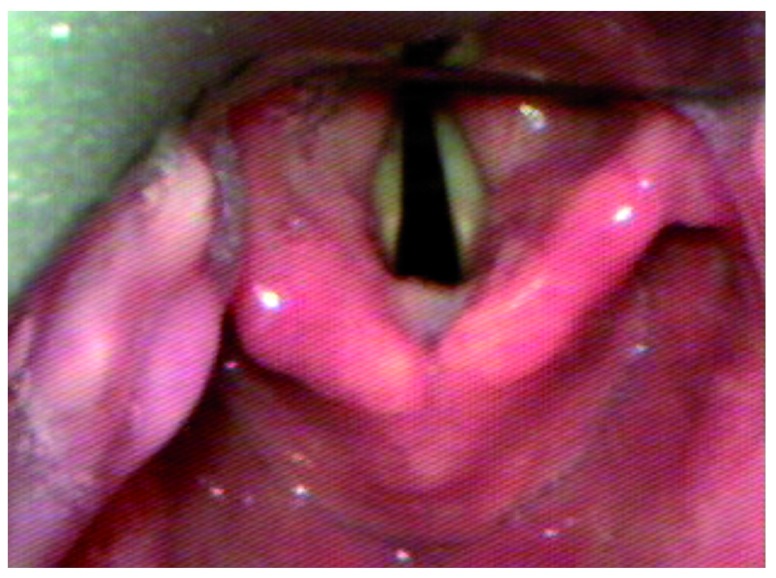
An acute-angle video laryngoscope is inserted deep, such that the epiglottis is lifted. A good laryngeal view is achieved.

One potential cause of difficult tube passage results when an acutely curved video laryngoscope is inserted too deep. Figure 1– Figure 3 display this phenomenon in which an adequate laryngeal view is achieved, but the glottis is lifted too anteriorly to permit tube passage. When the laryngoscope is withdrawn so that the tip of the blade is in the vallecula, the glottis falls into a more posterior position and is better aligned for tube passage. While intubating nasally, the use of adjunct techniques such as Magill forceps may be very useful to overcome the acute curvature of devices such as the GlideScope of C-MAC with D-blade.

**Figure 2.**
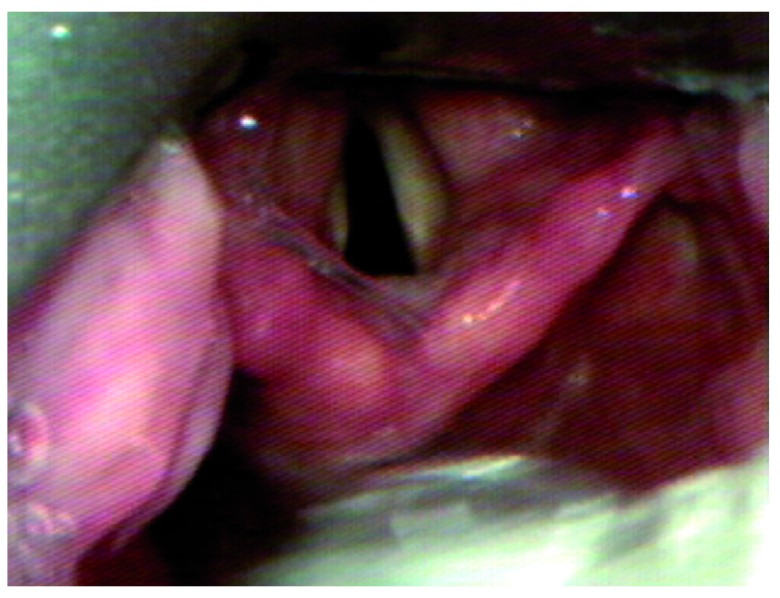
With the laryngoscope in this position, the glottis is in a very anterior position and the tube cannot reach the glottic inlet.

**Figure 3.**
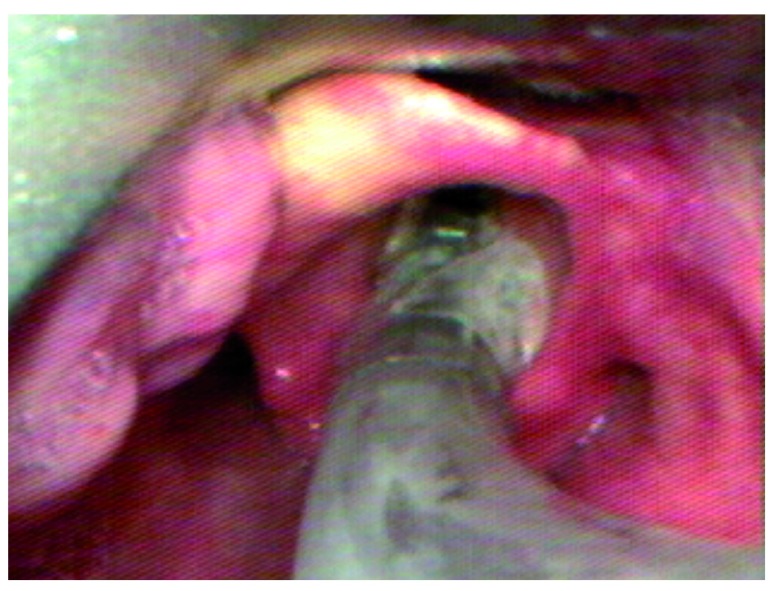
When the laryngoscope is withdrawn such that the tip of the blade is in the vallecula, the glottis falls into a more posterior position and becomes aligned with the tube trajectory.

## Video laryngoscopy: stress

Many authors believe that video laryngoscopy causes less patient stress than direct laryngoscopy. Indeed, less suspension pressure and extension of the cervical spine may be necessary to achieve an adequate laryngeal view. However, well-designed clinical trials have been inconsistent in demonstrating that video laryngoscopy is associated with less cervical traction than direct laryngoscopy when manual in-line stabilization is applied ^30^.

## Video laryngoscopy: trauma

Video laryngoscopy appears to expose the patient to different kinds of iatrogenic trauma compared with direct laryngoscopy. Reports continue to warn that pharyngeal injury is a problem with video laryngoscopy, although the incidence appears to be low. In particular, there are reports of tracheal tubes passing through the pharynx during video laryngoscopy ^31^. In fact, the operator, not the device, is responsible for this injury. However, despite more widespread knowledge of this potential problem, this complication occurs even in experienced clinical hands. Caution is warranted when advancing a tracheal tube through the pharynx during video laryngoscopy, especially when visual attention may be distracted from the patient. Specifically, the provider needs to watch the tube pass into the mouth and make the turn toward the larynx prior to focusing further attention on the video screen.

## Video laryngoscopy: future directions

Future research is poised to address some new questions. In particular, very few studies have compared video laryngoscope types and designs to determine the ideal device characteristics. Some limited data suggest that devices with channeled components (i.e. with a preloaded tube) result in faster and easier intubation than non-channeled devices ^32^. Furthermore, questions surrounding blade design continue to arise. For example, video-assisted direct laryngoscopes (i.e. Macintosh blade design) have the potential benefit of familiarity, simple tube passage, and narrow blade profile. On the other hand, acutely curved blade designs may further augment laryngeal view for the anterior airway beyond what may be achieved with a video-assisted direct laryngoscope. Thus far, studies have not shown a clear difference in success rate between various blade design types ^33, 34^.

## Conclusions

In summary, video laryngoscopy has established a permanent role in difficult airway management. Future investigations will help guide the clinical scenarios for use, algorithm approaches, and device designs. It is less clear whether video laryngoscopy will one day replace direct laryngoscopy. Currently, cost remains a barrier to such transitions for routine clinical care in the operating room. In other environments, that transition has occurred already on the basis of observational data, suggesting a reduction in intubation attempts.
